# Increased male-induced harm in response to female-limited selection: interactive effects between intra- and interlocus sexual conflict?

**DOI:** 10.1098/rspb.2023.0140

**Published:** 2023-04-26

**Authors:** Ana Ángela Romero-Haro, Lorenzo Pérez-Rodríguez, Barbara Tschirren

**Affiliations:** ^1^ Centre for Ecology and Conservation, University of Exeter, Penryn TR10 9FE, UK; ^2^ Instituto de Investigación en Recursos Cinegéticos (IREC), CSIC-UCLM-JCCM, Ronda de Toledo 12, 13005 Ciudad Real, Spain

**Keywords:** mating costs, life-history trade-offs, oxidative stress, traumatic mating, maintenance of genetic variation, indirect costs of reproduction

## Abstract

Interlocus sexual conflict (IRSC) occurs because of shared interactions that have opposite effects on male and female fitness. Typically, it is assumed that loci involved in IRSC have sex-limited expression and are thus not directly affected by selective pressures acting on the other sex. However, if loci involved in IRSC have pleiotropic effects in the other sex, intersexual selection can shape the evolutionary dynamics of conflict escalation and resolution, as well as the evolution of reproductive traits linked to IRSC loci, and vice versa. Here we used an artificial selection approach in Japanese quail (*Coturnix japonica*) to test if female-limited selection on reproductive investment affects the amount of harm caused by males during mating. We found that males originating from lines selected for high female reproductive investment caused more oxidative damage in the female reproductive tract than males originating from lines selected for low female reproductive investment. This male-induced damage was specific to the oviduct and not found in other female tissues, suggesting that it was ejaculate-mediated. Our results suggest that intersexual selection shapes the evolution of IRSC and that male-induced harm may contribute to the maintenance of variation in female reproductive investment.

## 1. Introduction

Selection often acts in different ways on males and females, resulting in divergent sex-specific optima in morphology, behaviour, physiology and life history [1,2]. Such sex-specific optima can lead to sexual conflict when alleles at a shared locus have antagonistic effects on male and female fitness (intralocus sexual conflict, IASC), or when interests of males and females diverge during reproductive interactions (interlocus sexual conflict, IRSC) [1,3]. The latter can promote the evolution of sexually antagonistic male strategies, such as coercive behaviours or toxic ejaculates, which benefit male reproductive success to the detriment of their female partner. A classic example of such an antagonistic male strategy is seminal fluid proteins in fruit flies (*Drosophila melanogaster*), which increase female egg production and sperm storage, but come at the cost of higher female mortality [4,5]. IRSC can result in sexually antagonistic coevolution (i.e. ‘sexual arms-race’) of male and female adaptations and counteradaptations and thus accelerate evolutionary change, whereas IASC can constrain evolution because of competing male- and female-specific selective forces acting on jointly expressed genes [6–9] (but see [10]). Given the different modes of operation, and different evolutionary consequences, IASC and IRSC have traditionally been considered as separate forces. Indeed, it is typically assumed that loci involved in IRSC have sex-limited expression and are thus not directly affected by selective pressures acting on the other sex [3,11]. However, if loci involved in IRSC have pleiotropic effects in the other sex, IASC could influence the evolutionary dynamics of IRSC escalation and resolution, and vice versa [11]. However, to date the role of intralocus conflict in the evolution of IRSC has received little attention.

Costs of reproduction are a key tenet of life-history theory [12,13]. Traditionally, researchers have focused on *direct* costs of reproduction, such as reduced self-maintenance as a consequence of increased resource allocation to reproduction, or increased physiological damage generated by increased reproductive effort [14,15]. However, costs can also be *indirect*, for example, in the form of male-induced harm during mating. If, as outlined above, loci associated with such male-induced harm have pleiotropic effects on female reproductive function, such indirect costs of reproduction can shape the evolution of life histories.

Unlike in invertebrates where the conflict potential of ejaculate components is well documented, this is much less the case in vertebrates. Indeed, studies in humans and laboratory rodents have traditionally considered ejaculate-induced changes in female physiology as an adaptive process that promotes healthy offspring development that benefits both males and females [16–19], largely neglecting the conflict potential associated with such processes. Understanding the conflict potential of ejaculate-mediated effects, and their genetic links to other reproductive traits and functions, can thus contribute to an integrative understanding of the costs of reproduction and provide insights into the constraints that shape life-history evolution.

Here we use an artificial selection approach in a precocial bird, the Japanese quail (*Coturnix japonica*) [20], to test (i) if there is evidence for ejaculate-induced harm to female partners and (ii) if sex-limited selection on female reproductive investment shapes the severity of harm caused by males (i.e. pleiotropic effects of loci linked to female reproductive function on IRSC). We have previously shown that female-limited selection on reproductive investment has correlated effects on male reproductive success, with males from lines selected for high female reproductive investment (H-line) siring more offspring than males from lines selected for low female reproductive investment (L-line) [21]. This pattern was observed in both a competitive mating situation (i.e. including male–male competition and female mate choice) and a non-competitive mating situation (i.e. excluding male–male competition and female mate choice) [21], suggesting that the male reproductive advantage was ejaculate-mediated, rather than caused by increased dominance or attractiveness [21]. We predict that if increased female reproductive investment is genetically linked to increased male-induced harm (i.e. pleiotropic effects on IRSC [11]), levels of physiological damage in females mated to an H-line male will be higher than levels of damage in females mated to an L-line male. Furthermore, if this effect is ejaculate-mediated, we predict that increased damage will be specifically found in the female reproductive tract, but not in other tissues.

## 2. Methods

### (a) Selection lines and breeding design

The study was conducted in a captive population of Japanese quail (*Coturnix japonica*) artificially selected for high (H-line) and low (L-line) maternal egg investment. Replicated selection lines were established as described in Pick *et al.* [20]. In brief, the top 25% of females producing the largest and smallest eggs relative to their body size, respectively, were selected for breeding in the first generation. In the subsequent generations, the 50% females producing the largest and smallest eggs were selected, respectively. In each generation, two sons and two daughters from each selected pair were used for the next breeding round. Breeding pairs were randomly allocated within line replicates and mating between relatives was avoided throughout the selection experiment. Note that unlike in experimental evolution experiments, this artificial selection approach does *not* provide potential for sexually antagonistic coevolution, and all correlated responses in males must thus be a direct consequence of female-limited selection on pleiotropic loci (see also Artificial selection approach in the electronic supplementary material).

After four generations of selective breeding, the H- and L-lines differed in egg size by more than 1 s.d., but they did not differ in the number of eggs laid [20]. We will refer to both females selected for low reproductive investment as well as males from the low female reproductive investment lines as L-line birds, and both females selected for high reproductive investment and males from the high female reproductive investment line as H-line birds.

For this experiment, both L-line and H-line females were randomly paired with either an L-line or H-line male (2 × 2 design; female / male pairs: *N* = 11 L / L, *N* = 10 L / H, *N* = 10 H / H, *N* = 10 H / L), and kept in breeding cages for two weeks (see [20] for a detailed description of the breeding and husbandry conditions). Eggs were collected every morning and weighed to the nearest 0.01 g. Three females (1 L / L, 1 H / H, 1 H / L) were not reproductively active (i.e. laid no eggs) and were thus removed from the analyses. There was no difference in the age of females between treatment groups (mean ± s.e.: L / L: 517 ± 105 days; L / H: 404 ± 58 days; H / H: 549 ± 111 days; H / L: 521 ± 107 days; all *p* > 0.445) or in the age of the males (L / L: 801 ± 60 days; L / H: 724 ± 60 days; H / H: 649 ± 45 days; H / L: 708 ± 67 days; all *p* > 0.161).

All females were blood sampled (approx. 100 µl) from the brachial vein using heparinized capillary tubes just before they were placed in the breeding cage. Blood samples were stored at 4°C until centrifugation (5 min at 20°C and 2000*g*) within 4 h. Plasma was then separated and frozen at −80°C until analysis. After breeding, all females were euthanized and tissue samples from the oviduct (isthmus), the liver and the spleen were snap frozen on dry ice and then stored at −80°C until analysis. All samples were obtained within a single session for all birds. All procedures were conducted under licenses provided by the Veterinary Office of the Canton of Zurich, Switzerland (permit numbers 195/2010; 14/2014; 156) and the ethical committee of the University of Exeter (permit eCORN002475).

### (b) Quantification of lipid oxidative damage

Oxidative damage is the result of non-alleviated oxidative stress, and lipids are a main target of such damage [22,23]. To quantify lipid oxidative damage in the female reproductive tract (oviduct), we measured levels of malondialdehyde acid (MDA), one of the endpoint molecules in the lipid peroxidation cascade. MDA is a commonly used marker of oxidative damage [22,24–27]. It is an extremely toxic and mutagenic molecule with high reactivity, interacting with DNA and proteins and damaging them [22,28]. High MDA levels have been related to numerous illnesses in humans (review in [29]), as well as lower fitness in non-human animals [24–27].

MDA quantification was performed using HPLC following the protocol of [30] with modifications by [31] (described in detail in the electronic supplementary material and in [32]). In addition to MDA measures in the oviduct, we also quantified MDA concentrations in the females' liver and spleen to verify that male-induced oxidative damage is specific to the reproductive tract, rather than systemic. Pre-breeding plasma MDA was quantified to test for potential differences in oxidative damage among females before the start of the experiment. One liver, one spleen and two oviduct samples were lost during handling, resulting in slightly lower sample sizes for some comparisons. All samples of a given tissue were analysed in a single session. Additional analyses of antioxidant activity in female tissues and plasma are presented in the electronic supplementary material.

### (c) Statistical analyses

First, we used linear models to test for an effect of male line, female line, their interaction and line replicate on levels of oxidative damage (MDA) in the females’ oviduct. To verify that these effects are specific to the reproductive tract rather than systemic, we ran the same analysis for female liver and spleen.

Second, we tested if male line origin affects female reproductive output. Quail are continuous layers and do not produce a traditional clutch. We therefore used the number of eggs a female laid over the 14-day breeding event (i.e. did/did not produce an egg on a given day) as a proxy for fecundity in a generalized linear model with a quasibinomial error structure. A quasibinomial model was used instead of a binomial model because of overdispersion. A linear model was used for mean egg mass. Male line, female line, their interaction and line replicate were included as fixed effects in the models. Significance of predictors was determined using the *car* package [33].

Normality of the residuals of linear models was confirmed by visual inspection and Shapiro–Wilk tests. All statistical analyses were performed in R version 4.1.2 [34]. Additional analyses of pre-breeding phenotypic traits, antioxidant activity in different tissues, effects of pre-breeding oxidative stress levels and body mass on post-breeding MDA levels in different tissues, effects of reproductive output on post-breeding MDA levels in different tissues, and correlations among oxidative stress markers within and across tissues are presented in the electronic supplementary material. Data and code are deposited in the Dryad Digital Repository: https://doi.org/10.5061/dryad.98sf7m0nn [35].

## 3. Results

### (a) Oxidative damage in reproductive and non-reproductive tissues

**Figure 1.**
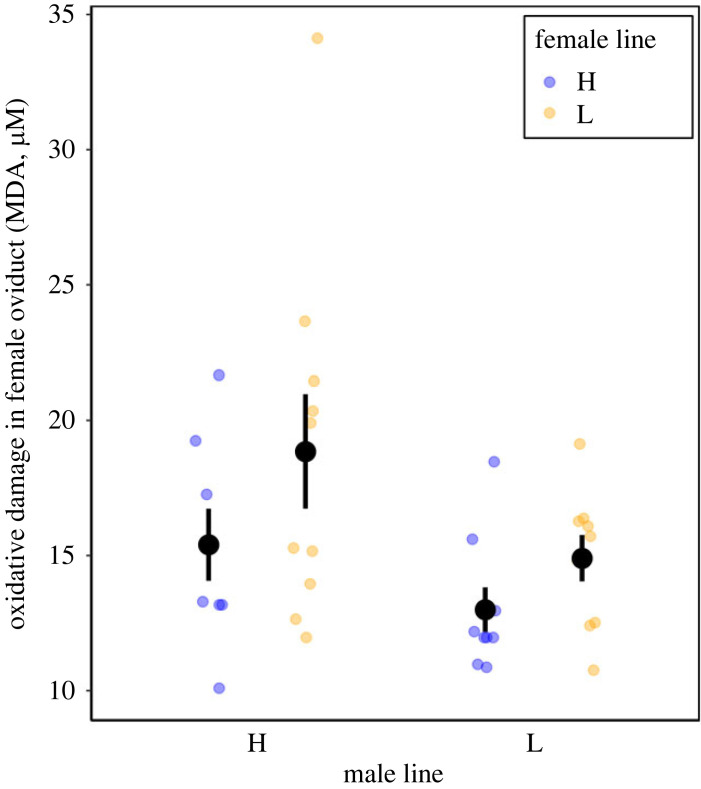
Effect of male line and female line on levels of oxidative damage to lipids (MDA) in the females' oviduct. Females mated to an H-line male had higher levels of damage in their oviduct than females mated to an L-line male. Means ± 1s.e. are shown. Dots represent raw data. H-line females are shown in light grey and L-line females in dark grey (online version in colour). Results did not change when removing the extreme value measured in an L-line female mated to an H-line male (electronic supplementary material, figure S1).

Females experimentally paired with an H-line male had significantly higher levels of lipid oxidative damage (MDA) in their oviduct than females paired with a L-line male (mean ± s.e.: 17.2 ± 1.02 µM, *n* = 18 and 14.0 ± 1.01 µM, *n* = 18, respectively), independent of their own line origin (table 1, figure 1). This effect of male line was specific to the oviduct and was not observed in the females' liver or spleen (electronic supplementary material, table S1 and figure S1). The effect was robust to the removal of an extreme value from the analysis (electronic supplementary material, figure S2).

**Table 1.** Effects of male line, female line, their interaction and line replicate on oxidative damage (MDA) levels in the females' oviduct (µM).

	estimate	s.e.	*F*	d.f.	*p*
intercept	14.500	1.685			
male line (L)	−2.302	2.096	5.021	1, 31	*0.032*
female line (L)	3.449	2.044	3.178	1, 31	0.084
replicate (2)	1.805	1.441	1.569	1, 31	0.220
male line (L) × female line (L)	−1.747	2.886	0.366	1, 31	0.549

No differences in female pre-breeding body mass, pre-breeding plasma MDA or pre-breeding plasma total antioxidant status were observed with respect to male line (electronic supplementary material, table S2), and including these pre-breeding measures into the model did not change the male line effect on oxidative damage in the females’ oviduct (electronic supplementary material, tables S3 and S4). No effect of male line on post-breeding measures of antioxidant activity in female reproductive or non-reproductive tissues was observed (electronic supplementary material, tables S5 and S6).

No effect of a female's own line on oxidative damage in the oviduct was observed (table 1, figure 1). However, L-line females had higher levels of MDA in the spleen than H-line females (mean ± s.e.: 88.4 ± 4.09 µM, *n* = 19, and 76.0 ± 4.20 µM, *n* = 18, respectively; electronic supplementary material, table S1 and figure S1b). There were no significant correlations among markers of oxidative status across tissues (electronic supplementary material, table S7).

### (b) Reproductive output

The number of eggs laid by a female during the reproductive event was not affected by male line, female line or their interaction (table 2). Egg mass was not significantly affected by male line or the interaction between female and male line (table 2). As expected, L-line females laid significantly smaller eggs than H-line females (mean ± s.e.: 10.9 ± 0.145 g, *n* = 20, and 12.0 ± 0.153 g, *n* = 18, respectively; table 2). Neither the number of eggs laid during the reproductive event, nor mean egg mass were significantly associated with levels of oxidative damage in the females' oviduct or other tissues (electronic supplementary material, table S8).

**Table 2.** Effects of male line, female line, their interaction and line replicate on female reproductive output. Note that estimates for the number of eggs laid are presented on a logit scale.

	estimate	s.e.	*F*	d.f.	*p*
**number eggs laid**					
intercept	1.177	0.356			
male line (L)	0.477	0.484	0.045	1, 33	0.834
female line (L)	0.787	0.498	0.402	1, 33	0.531
replicate (2)	−0.218	0.341	0.407	1, 33	0.528
male line (L) × female line (L)	−1.113	0.692	2.642	1, 33	0.114
**mean egg mass (g)**					
intercept	11.944	0.238			
male line (L)	0.378	0.309	0.560	1, 33	0.460
female line (L)	−0.968	0.301	30.434	1, 33	*<0.001*
replicate (2)	−0.129	0.213	0.364	1, 33	0.550
male line (L) × female line (L)	−0.416	0.426	0.955	1, 33	0.336

## 4. Discussion

Our study provides experimental evidence for male-induced oxidative damage in the female reproductive tract in a precocial bird and shows that increased female reproductive investment is genetically linked to increased male-induced harm. Selection on increased female reproductive investment thus appears to generate a sexual conflict, by generating males that cause increased harm to females during mating. Such indirect costs of reproduction may constrain the evolution of female reproductive investment and contribute to the maintenance of variation in reproductive investment in populations.

We imposed selection on a female-limited trait (egg investment) in our experiment, and the study design prevented male–female coevolution. Correlated responses in males, including the level of harm caused to their partner during mating, must thus be the consequence of pleiotropic effects of genes selected in females. It suggests that IRSC and IASC may not be separate forces (as traditionally believed [3,11]), but may indeed be closely interlinked. Such interactions have important implications for conflict resolution and escalation, as well as the evolution of traits linked to sexually antagonistic male strategies.

The effect of male line origin on levels of oxidative damage in the female was restricted to the reproductive tract and not found in other tissues, suggesting that it was ejaculate-mediated. In laboratory mice, it has recently been shown that a male's diet affects seminal fluid immune-regulatory activity, leading to changes in inflammatory responses in the female reproductive tract [36]. If the amount and/or composition of the ejaculate of males from the divergent selection lines differ, either because they had more (H-line) or less (L-line) resources available during prenatal development or because ejaculate composition is genetically linked to female reproductive investment [21,37,38], then such effects could explain how selection on a female-limited trait can generate sexual conflict (see also [39]). Similarly, the foamy substance that is produced by the proctodeal gland of quail males and transferred to females during copulation has been shown to contain prostaglandins [40]. Prostaglandins induce contractions in the female reproductive tract that increase the mobility of sperm within the oviduct [40], but also stimulate inflammatory responses in the female reproductive tract (reviewed in [41]). If males from the divergent lines differ in the amount or composition of proctodeal gland foam they transfer, this could generate the observed effect [42,43]. Finally, oxidative damage generated through female reproductive tract–ejaculate interactions may underlie the observed patterns [44]. Sperm storage tubules (SST) in the female reproductive tract contain a complex antioxidant system to prevent and repair damage to spermatozoa caused by peroxidation (see [45] and references therein), and they secrete diverse metabolites, such as fatty acids, to maintain the functional integrity of stored sperm (see [46] and references therein). Recently, it has been demonstrated in chicken that these secreted lipids are highly vulnerable to peroxidation, and that females generating more fatty acids in their SST to protect stored sperm show higher levels of oxidative damage (MDA) in their oviducts [46]. Sperm of H-line males may be more susceptible to peroxidation and/or require a higher SST fatty acid production for maintenance, potentially explaining the higher levels of oxidative damage in the female's reproductive tract when mated to an H-line male. Indeed, a higher proportion of polyunsaturated fatty acids in the membrane of fowl spermatozoa has been related to an enhanced male fertility [47], but, at the same time, also to a higher susceptibility to oxidative damage (reviewed in [48,49]). Higher fertility is observed in H-line males compared to L-line males [21], making this a plausible scenario.

Irrespective of the exact mechanism underlying the observed male line effect, evidence from other studies suggests that increased levels of oxidative damage in the reproductive tract may have negative fitness consequences for the female. Indeed in humans, high levels of oxidative stress in the female reproductive tract are related to diverse disorders, such as infertility, miscarriages, preeclampsia, fetal growth restrictions and diverse embryopathies, leading to maternal and offspring morbidity and mortality [50–52]. Similarly, oxidative damage in the female reproductive tract negatively affects fecundity in laboratory rodents [53,54]. Increased MDA levels have also been associated with the ageing process [55] and the occurrence of certain diseases, such as cancer, Alzheimer's disease and diabetes (reviewed in [29]). Finally, negative associations between MDA levels and fitness have repeatedly been found in natural populations [24,26,27]. However, whereas this previous work provides evidence for a negative association between oxidative damage and fitness, we cannot directly demonstrate such fitness costs in our study because females had to be sacrificed for the quantification of physiological damage in the reproductive tract. Following a cohort of females throughout their life to quantify the long-term costs of male-induced harm for female reproductive success and lifespan would thus be a fruitful next step to directly quantify such fitness costs in our system.

Increased harm to females might directly increase male reproductive success, as for example observed in *Drosophila*, where accessory gland products increase female egg-laying rates at the expense of female survival [5]. However, we found no evidence that females mated to H-line males produced more or larger eggs (see also [56,57]), making this an unlikely scenario. Alternatively, changes in ejaculate composition might directly or indirectly (via modifying physiological responses in the female reproductive tract, see above) benefit males in a sperm competition context, with increased harm in the female reproductive tract merely reflecting collateral damage [58,59]. Sperm competition experiments, and tests of sperm function when sperm is exposed to ejaculates from H- versus L-line competitors, would be interesting next steps to directly test these hypotheses.

Whereas we observed a strong male line effect, no effect of a female's own line origin on levels of oxidative damage in the reproductive tract was observed. Furthermore, oxidative damage in the female reproductive tract was not associated with the size or number of eggs a female laid. Interestingly, L-line females had significantly higher levels of oxidative damage in the spleen, an important immune organ [60], than H-line females. We have previously shown that the immune system of L-line females is upregulated compared to H-line females [61], and higher levels of oxidative damage might be a direct consequence of this upregulation [42,43]. Together, these findings suggest that increased oxidative damage is an indirect (i.e. male-induced) rather than direct cost of reproductive investment in our system.

Within a female, levels of oxidative damage and antioxidant activity were not, or only weakly correlated within and across tissues. This highlights that patterns of oxidative stress can be highly tissue specific, leading to within-body mosaics of damage accumulation. This not only has implications for our understanding of life-history evolution, but also questions the validity of using blood samples to quantify the overall oxidative status of an individual (e.g. [62]). Indeed, it is often assumed that oxidative stress in plasma accurately reflects oxidative stress in other tissues [63,64]. This would require oxidative stress levels in different parts of the body to be correlated (i.e. systemic oxidative stress). However, as we show here, this is not necessarily the case.

In conclusion, our study provides experimental evidence for a genetic link between female reproductive investment and male-induced damage in the female reproductive tract, suggesting that intralocus conflict may shape the evolutionary dynamics of IRSC, and that IRSC may constrain the evolution of reproductive investment. It suggests that the distinction between IASC and IRSC may not be as clear as traditionally thought [3,11], and that these two processes can indeed be interlinked.

## Data Availability

Data and code are deposited in the Dryad Digital Repository: https://doi.org/10.5061/dryad.98sf7m0nn [35]. Additional data are provided in the electronic supplementary material [65].
